# Influence of Interlayer Connection Type on the Flexural Performance and Failure Mechanisms of Three-Layer Timber Composites

**DOI:** 10.3390/ma19214562

**Published:** 2026-10-29

**Authors:** Izabela Burawska, Magdalena Omelańczuk

**Affiliations:** 1 Faculty of Forestry and Wood Sciences, Warsaw University of Life Sciences—SGGW, Nowoursynowska 159, 02-776 Warsaw, Poland; 2 Faculty of Wood Technology, Warsaw University of Life Sciences—SGGW, Nowoursynowska 159, 02-776 Warsaw, Poland

**Keywords:** Scots pine, polyurethane adhesive, wooden connectors, adhesive-free assembly, annual-ring orientation

## Abstract

The development of adhesive-free layered timber composites requires effective mechanical connections capable of ensuring sufficient composite action between individual layers. This study investigated the effects of interlayer connection type (polyurethane adhesive bonding and wooden dowels) and annual-ring orientation of inner layer on the flexural performance and failure mechanisms of three-layer Scots pine (*Pinus sylvestris* L.) composites. Four configurations were evaluated through bending tests, considering apparent modulus of elasticity (MOEapp), apparent modulus of rupture (MORapp), apparent bending stiffness (EIapp), energy absorption capacity (EC), and density. A two-way analysis of variance was performed to assess the effects of both experimental factors and their interaction. Connection type was the dominant factor influencing mechanical performance. Adhesive-bonded composites exhibited MOEapp values of 8.35–9.82 GPa and MORapp values of 134–141 MPa, whereas dowel-connected composites achieved 1.36–1.40 GPa and 71–74 MPa, respectively. On average, dowel connections were associated with an approximately 85% reduction in MOEapp and a 47% reduction in MORapp compared with adhesive bonding. Conversely, dowel-connected specimens exhibited approximately 2.1–2.7 times greater energy absorption capacity. Annual-ring orientation of the boards forming the composite’s middle layer had a statistically significant but relatively small effect on MOEapp, while its influence on the remaining mechanical parameters was not significant. No statistically significant differences in density were identified among the investigated configurations. Adhesive-bonded specimens predominantly exhibited timber failure without visible bond-line damage, whereas dowel-connected specimens showed more complex failure behaviour involving timber rupture, connector damage, and interlayer displacement. No clear differences in failure mechanisms were observed in relation to the annual-ring orientation of the core layer. The findings highlight the dominant role of interlayer connection type in the global flexural response of layered timber composites and indicate the need for further investigation of connector stiffness and partial composite action in adhesive-free systems.

## 1. Introduction

Engineered wood products, most notably cross-laminated timber (CLT) and layered timber composites, are at the forefront of contemporary sustainable construction [1,2]. Their increasing use in both residential and commercial projects is attributed to high mechanical performance, low environmental impact, and prefabrication, which together can reduce construction time and lower structural weight compared to conventional solutions such as reinforced concrete and steel [3,4]. The increasing adoption of these systems is closely related to global strategies aiming to reduce embodied carbon, promote renewable resources, and support circular economy principles in the built environment [5,6,7,8,9]. However, structural performance of engineered timber elements depends not only on the properties of the raw material but also on interlayer connections and the arrangement of individual components.

The method used to connect individual layers is a key factor influencing the load-bearing capacity, stiffness, and structural behaviour of layered timber composites. The dominant approach in industrial practice is adhesive bonding, employing synthetic resins such as polyurethane (PU), melamine-urea-formaldehyde (MUF), or phenol-resorcinol-formaldehyde (PRF), which enable effective composite action and efficient stress transfer between layers [3,10,11]. Such systems offer high stiffness and mechanical efficiency, and their design methodologies are well established in engineering standards. However, adhesive-based solutions introduce challenges related to end-of-life processing, recyclability, and environmental impacts, particularly in light of concerns about volatile organic compound (VOC) emissions and the fossil origin of most commercial adhesives [4,10,12]. In the context of ongoing research into green chemistry and renewable binders, it is important to clarify that the present study utilizes a conventional polyurethane adhesive, as this choice reflects prevailing industry usage and enables direct comparison with mechanical connection strategies.

Mechanical fasteners—such as dowels, screws, and nails—transfer loads between timber layers through discrete connection points rather than continuous bonding [12,13,14,15]. These systems, sometimes referred to as dowel-laminated timber (DLT) or nail-laminated timber (NLT), are particularly attractive in the context of circular economy objectives, as they may facilitate disassembly, reuse, and recycling of individual components at the end of a structure’s service life [4]. However, replacing continuous adhesive bonding with discrete mechanical connections may result in reduced global stiffness, altered failure modes, and increased sensitivity to moisture and long-term loading. Studies have shown that dowel-type connections may be susceptible to interlayer slip, rolling shear, and complex interactions between fastener geometry and wood anisotropy, especially under sustained or cyclic loads [12,13]. Consequently, hybrid systems and reversible connectors have been proposed to bridge the gap between structural efficiency and sustainability by combining partial adhesive bonding with mechanical fastening strategies [15,16].

Previous studies have investigated these mechanisms using different materials, connection geometries, and experimental approaches. Pereira et al. [17] examined dowelled cross-laminated timber panels manufactured from *Pinus elliottii* and *Pinus taeda*, focusing on the mechanical behaviour of wooden dowel connections and the evaluation of interlayer slip. Derikvand et al. [18] investigated three-layer dowel-laminated timber beams assembled using connectors manufactured from solid wood and recovered engineered wood products, demonstrating that connector properties significantly influence composite action and bending stiffness. Xu et al. [19] examined three-layer adhesive-free CLT beams connected with densified wood dowels and assessed the effects of dowel number and arrangement on bending strength and effective stiffness. Other investigations have directly compared mechanically connected and adhesively bonded timber elements. For example, Dourado et al. [20] examined laminated pine beams incorporating wooden dowels with different insertion angles and compared their bending performance with that of glued reference beams. More recently, Aloisio et al. [6] investigated the bending stiffness of wooden-dowelled CLT panels under different moisture conditions and compared their structural response with that of conventionally glued CLT. These investigations established that connection characteristics and interlayer slip play a major role in the mechanical performance of layered timber systems.

Although the above studies provide substantial knowledge of connector efficiency and composite action, their experimental objectives primarily concerned connector material, geometry, arrangement, or moisture-dependent behaviour. The present study addresses a different aspect by considering the combined influence of connection type and annual ring orientation within the same three-layer composite configuration. This distinction is relevant because the anatomical orientation of wood may affect local deformation and stress transfer, while the extent to which these effects are reflected in global bending performance depends on the mechanical interaction between adjacent layers.

Wood is an orthotropic material with different mechanical properties in the longitudinal, radial, and tangential directions. Consequently, annual growth ring orientation may affect stiffness, strength, and deformation behaviour, particularly when loading involves stresses perpendicular to the grain or shear deformation. Previous investigations have demonstrated the influence of annual ring structure on the transverse mechanical properties and rolling shear behaviour of Scots pine wood [21]. However, the effect of annual ring orientation on the global bending performance of a layered composite must also be considered in relation to its internal configuration and the degree of interlayer stress transfer. In continuously bonded elements, deformation compatibility between layers is promoted by the adhesive bond, whereas mechanically connected elements may additionally experience local connector deformation and interlayer slip. These differences provide a basis for investigating whether annual ring orientation affects the bending response to a similar extent in adhesive-bonded and dowel-connected systems.

The remaining research question is therefore not whether adhesive bonding provides greater composite action than mechanical connections, as this behaviour has already been established, but how the relative influence of connection type and annual ring orientation can be evaluated within an equivalent composite configuration. In particular, a controlled experimental comparison is needed to determine whether changes in annual ring orientation measurably affect bending stiffness and strength when the interlayer connection changes from continuous adhesive bonding to discrete wooden dowels. The associated failure mechanisms are also relevant, as they provide information on the relationship between load-transfer efficiency and the structural response of individual composite variants.

Accordingly, the aim of this study was to investigate the effects of interlayer connection type and annual ring orientation on the flexural performance and failure mechanisms of three-layer Scots pine (*Pinus sylvestris* L.) composites. Four experimental variants were prepared by combining two connection systems—continuous polyurethane (PUR) adhesive bonding and smooth pine wood dowels—with two annual ring orientations of the inner layers. Matched timber elements were used, and their initial modulus of elasticity was determined to obtain comparable material properties across the experimental variants. The study evaluated density, bending modulus of elasticity (MOEapp), modulus of rupture (MORapp), apparent bending stiffness, energy absorption capacity, and failure mechanisms under four-point bending. The specific contribution of the research lies in the controlled evaluation of the relative importance of interlayer connection type and annual ring orientation within an equivalent three-layer configuration. Rather than introducing a new connection technology, the study provides experimental evidence of how these two factors relate to global bending performance and failure behaviour, thereby complementing previous research focused primarily on connector characteristics and composite action.

## 2. Materials and Methods

The experimental program was conducted using Scots pine (*Pinus sylvestris* L.) timber obtained from Kleniewski Tartak, Bondyrz, Poland. According to information provided by the supplier, the lumber used for specimen preparation originated from the Mazowiecko-Podlaska Forest Region of Poland. The timber was selected from commercially available sawn boards. Prior to specimen preparation, the timber was visually inspected, and boards exhibiting major defects, such as large knots, visible cracks, pronounced grain deviations, signs of biological degradation, or substantial geometric distortions, were excluded. This selection aimed to limit the influence of major wood defects on bending performance while retaining the natural variability of Scots pine timber.

The selected boards were machined to a final cross-section of 22 mm × 140 mm. Each 2000-mm-long board was then cut into two 1000-mm-long sections, referred to as twin elements, which were subsequently used as the upper and lower outer layers of the same composite beam. This approach was adopted to reduce differences in anatomical structure and mechanical properties between the tensile and compressive zones of the composite during bending.

Prior to composite fabrication, all twin boards were conditioned under controlled laboratory conditions (22 °C, 65% RH). Moisture content was determined using a MERLIN Technology EVO WW moisture meter (Merlin Technology GmbH, Tumeltsham, Austria). The mean moisture content of the timber was 9.3%, ranging from 8.7% to 9.6%. Mass was measured using a laboratory balance (RADWAG, Radom, Poland) with an accuracy of ±0.02 g, while dimensions (thickness, width, length) were determined using a digital caliper (Mitutoyo Corporation, Kawasaki, Japan), with each value recorded as the mean of three measurements. Density was calculated using the stereometric method.

**Figure 1:**
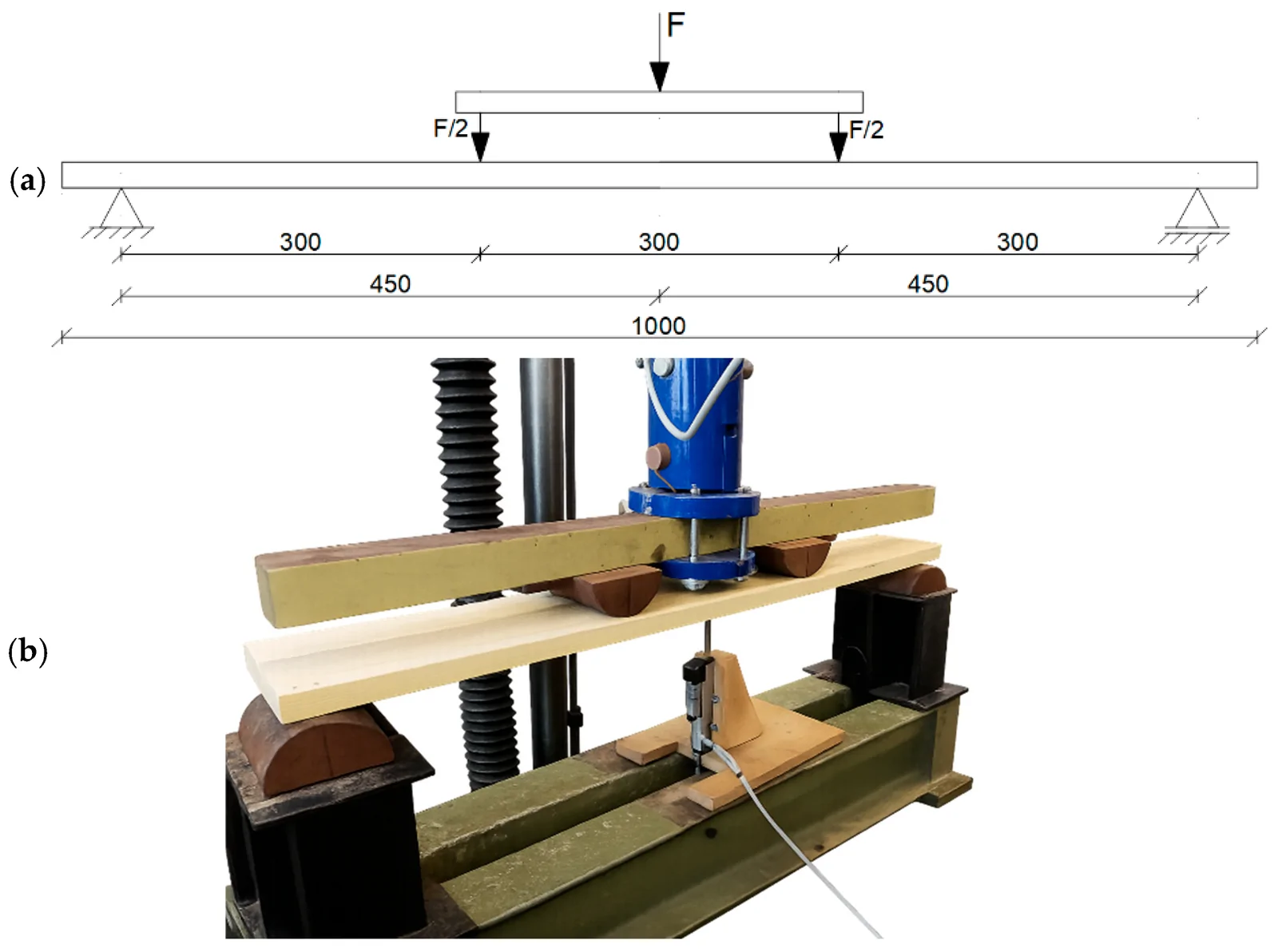
Load configuration during the four-point bending test: (**a**) schematic diagram (all dimensions in mm); (**b**) photograph of the test setup.

The modulus of elasticity of each individual outer lamella (MOE_lam) was determined by static four-point bending (Figure 1a,b), using a flatwise configuration and a loading arrangement based on EN 408:2012. The support span was 900 mm, and the distance between each support and the nearest loading point was 300 mm. Loading was applied within the elastic range at a displacement-controlled rate of 0.06 mm/s, with a maximum total applied force of 2 kN. After reaching the prescribed load, the specimens were unloaded to avoid damage before composite fabrication. Mid-span deflection was measured using Novotechnik TRS 75 (Novotechnik Messwertaufnehmer OHG, Ostfildern, Germany) displacement transducers (accuracy ±0.01 mm). The modulus of elasticity was calculated from the slope of the load–deflection curve within the linear elastic range using Equation (1):

(1) $$\mathrm{MOE}\_\mathrm{lam}=\frac{23(F_{2}-F_{1})L^{3}}{108bh^{3}(w_{2}-w_{1})}$$

where:

*F*_1_ and *F*_2_—total applied loads defining the selected linear range of the load–deflection curve (N);
*w*_1_ and *w*_2_—corresponding mid-span deflections (mm);
*L*—distance between supports (900 mm).
*b*—specimen width;
*h* —specimen height.

The resulting MOE_lam values were used to characterize the initial flexural stiffness of the timber elements and to allocate the twin outer lamellas to the four experimental variants. The allocation was performed to ensure comparable ranges and variability of MOE_lam values across the four groups, thereby minimizing potential differences in the initial mechanical properties of the outer layers. This approach was intended to reduce the influence of natural timber variability and enable a more reliable comparison of the effects of interlayer connection type and annual ring orientation of the transverse core.

**Figure 2:**
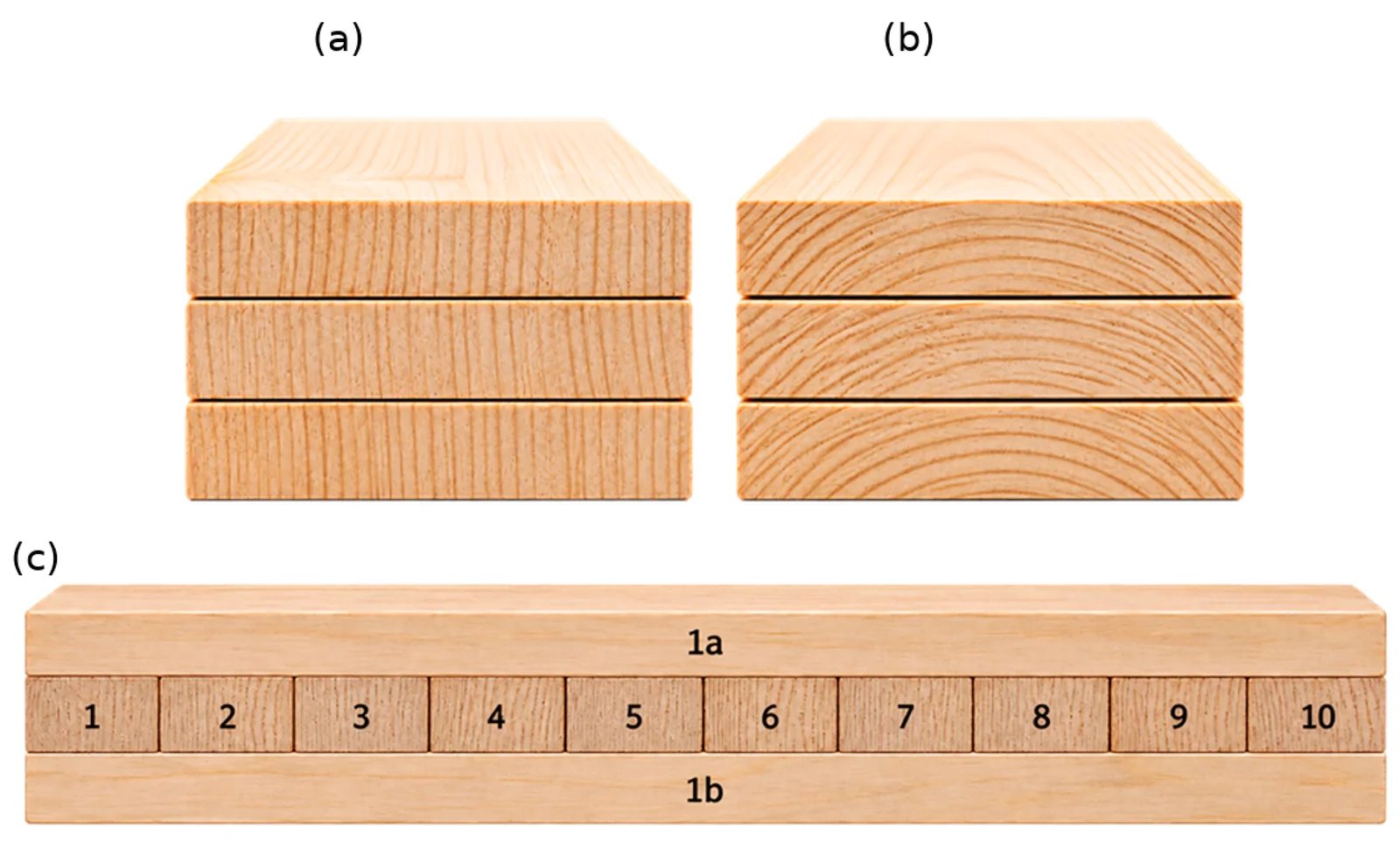
Annual ring orientation and arrangement of timber components in the three-layer composite: (**a**) standing annual rings; (**b**) lying annual rings; (**c**) arrangement of composite components, where 1a and 1b denote the outer layers (22 mm × 140 mm × 1000 mm), and 1–10 denote the transverse core lamellas (22 mm × 100 mm × 140 mm).

A separate set of Scots pine lamellas with nominal dimensions of 22 mm × 100 mm × 140 mm was prepared for the transverse core layer. The lamellas were characterized in terms of density and classified according to their annual ring orientation in the cross-section as either standing (vertical) or lying (horizontal) (Figure 2). Annual ring orientation was investigated exclusively in the core layer and constituted one of the two experimental factors.

Each three-layer composite beam consisted of two longitudinal outer lamellas (22 mm × 140 mm × 1000 mm) and a transverse core formed by ten consecutively arranged lamellas (22 mm × 100 mm × 140 mm) (Figure 2). The longitudinal grain direction of the outer layers was maintained in all experimental variants, whereas the annual ring orientation of the transverse core lamellas was varied according to the experimental design. All ten core lamellas within each composite beam had the same annual ring orientation, corresponding to the assigned experimental variant.

Two connection systems were investigated:

(1) Adhesive-bonded system, using polyurethane (PUR) adhesive to provide continuous bonding between layers.
(2) Dowel-connected system, using smooth pine dowels with a diameter of 16 mm to provide discrete mechanical connections between layers.

Four variants of three-layer composite beams were prepared (Table 1).

**Figure 3:**
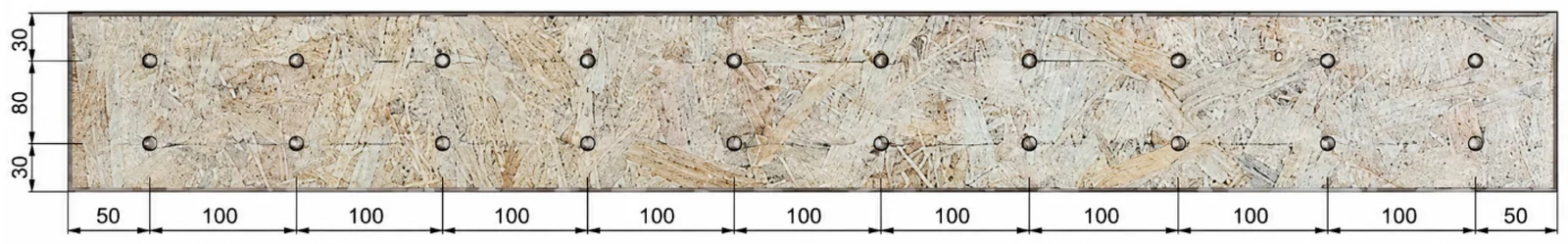
Drilling pattern used for dowel connections. All dimensions are in mm.

In Variants 1 and 2, layers were bonded using Chemolan B, version 45 (Interchemol, Oborniki Śląskie, Poland), a one-component, moisture-curing polyurethane (PUR) adhesive with a D4 water-resistance classification. The adhesive was applied manually using a brush to both bonding surfaces at a nominal spread rate of approximately 180–220 g/m^2^ to ensure full coverage. The open assembly time was 15 min, while the closed assembly time was approximately 20 min. The assembled composites were subsequently pressed in a cold press (Höfer Presstechnik GmbH, Taiskirchen im Innkreis, Austria) at a pressure of 0.4 N/mm^2^ (0.4 MPa) for 3 h. Following pressing, the bonded composites were conditioned for 72 h before mechanical testing. The adhesive-bonded specimens served as an experimental reference configuration with continuous interlayer bonding, allowing comparison with the mechanically connected composites. In Variants 3 and 4, the layers were connected mechanically using 20 smooth Scots pine wood dowels with a nominal diameter of 16 mm and a moisture content of approximately 8%. The wood grain was oriented longitudinally, parallel to the dowel axis. The dowels extended through the full thickness of the three-layer composite, connecting both longitudinal outer layers with the transverse core. Each dowel acted as a double-shear connector, with two potential shear planes located at the interfaces between the core and the outer layers. Boreholes with a nominal diameter of 15.5 mm were drilled through all three layers using a column drilling machine (Bosch PBD 40, Robert Bosch Power Tools GmbH, Stuttgart, Germany), providing a nominal diametral interference of 0.5 mm. The dowels were arranged according to the predefined drilling pattern, with nominal spacings of 100 mm and 80 mm in the respective directions. The distances between the dowels and the edges and ends of the composite beams are specified in Figure 3. The dowels were inserted using a manual press (Rodent, Pruszków, Poland) without adhesive, producing an entirely mechanically connected assembly.

**Figure 4:**
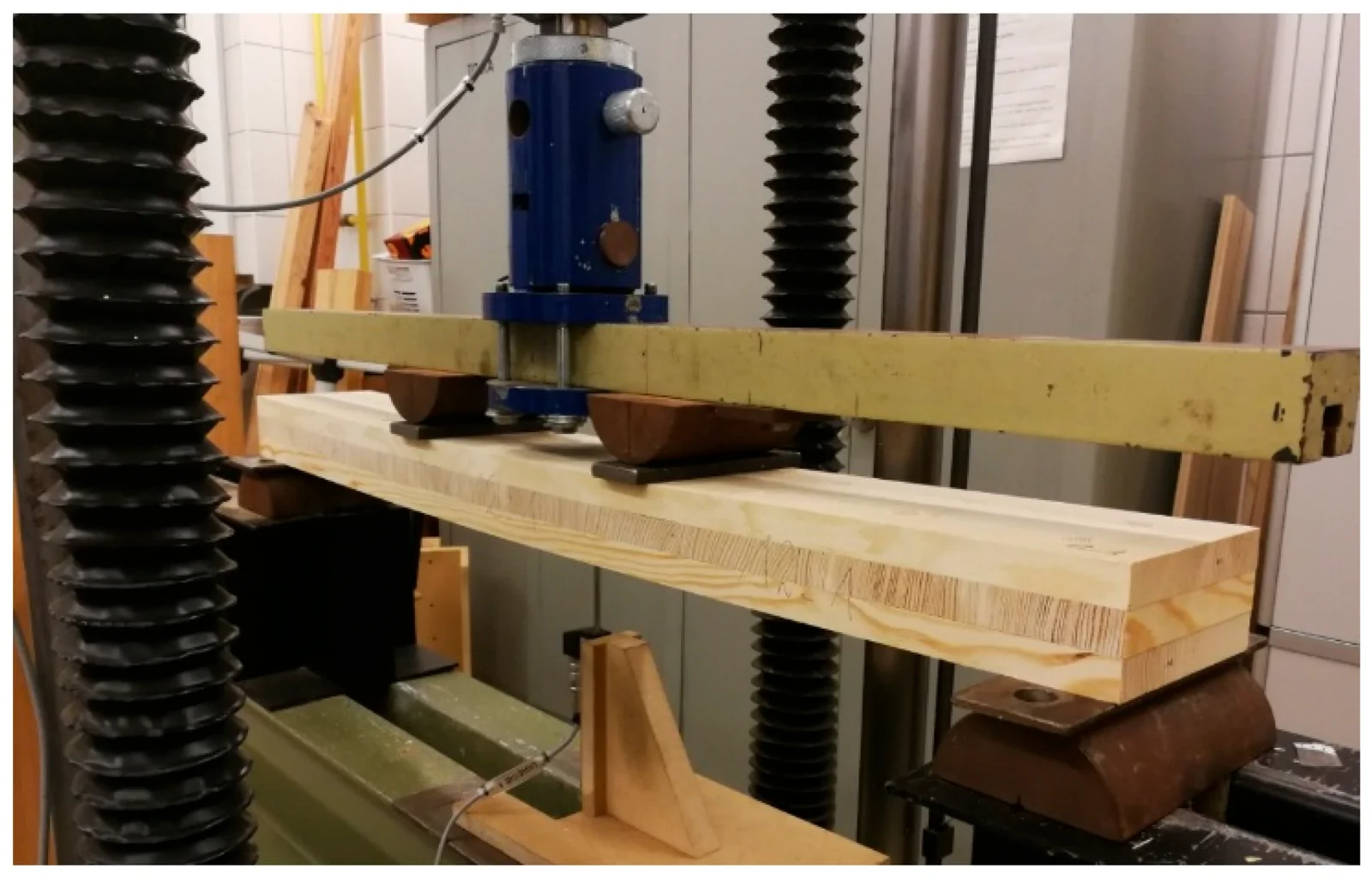
Composite beam during bending strength test.

Bending tests were conducted on all composite beams (six specimens per variant) The test configuration, including span and loading arrangement, is shown in Figure 4. Loading was applied monotonically under displacement control at a constant rate of 0.08 mm/s until failure. Mid-span deflection was measured using Novotechnik TRS 75 (Novotechnik Messwertaufnehmer OHG, Ostfildern, Germany) displacement transducer positioned at the centre of the span. Data acquisition was carried out at a sampling rate of 2 Hz.

The mechanical properties of the composite beams were determined from the global load–deflection response obtained during four-point bending. The apparent bending stiffness, EIapp, was calculated from the slope of the load–deflection curve within the linear elastic range, using the beam-deflection relationship for symmetrical four-point loading. The apparent global modulus of elasticity (MOEapp) was determined by dividing the apparent bending stiffness by the gross second moment of area of the composite cross-section. The apparent modulus of rupture (MORapp) was calculated from the maximum bending moment and the gross section modulus.

The calculated parameters characterize the global structural response of the assembled composites rather than the intrinsic mechanical properties of the constituent timber. This distinction is particularly important for dowel-connected specimens, in which global deflection may include contributions from interlayer slip, connector deformation, and rolling shear of the cross-oriented core.

The apparent global modulus of elasticity of the composite beams (MOEapp) was calculated using Equation (1), following the same loading geometry and calculation procedure as for the individual outer lamellas, but considering the overall cross-sectional dimensions of the assembled composite.

The apparent bending stiffness and apparent modulus of rupture were calculated using the following equations:

(2) $$\mathrm{EIapp}=\frac{(F_{2}-F_{1})a\left(3L^{2}-4a^{2}\right)}{48(w_{2}-w_{1})}$$

where:

EIapp —apparent bending stiffness (N·mm^2^);
a—distance between a support and the nearest loading point (300 mm).

(3) $$\mathrm{MORapp}=\frac{F_{\max}L}{bh^{2}}$$

where:

MORapp—apparent modulus of rupture (MPa);
*F_max_*—maximum total applied load recorded during the test (N).

The energy absorption capacity (EC) was determined by numerical integration of the recorded force–displacement curve using the trapezoidal rule. The integration was performed from the beginning of loading to the point at which the applied force decreased to 85% of the maximum load (F_max_) on the descending branch of the curve. In specimens exhibiting abrupt brittle failure, where the 85% F_max_ criterion could not be reliably identified due to the sudden loss of load-bearing capacity, integration was terminated at the last valid recorded data point preceding the abrupt failure.

The energy absorption capacity was calculated using Equation (4):

(4) $$\mathrm{EC}=\frac{1}{1000}\sum_{i=1}^{n-1}\frac{F_{i}+F_{i+1}}{2}(d_{i+1}-d_{i})$$

where:

EC is the energy absorption capacity (kJ);
F_i_ and F_i+1_ are the consecutive recorded values of the applied force (kN);
d_i_ and d_i+1_ are the corresponding displacement values (mm);
and n is the number of data points included in the numerical integration up to the defined endpoint.

The statistical analysis was designed to evaluate the effects of interlayer connection type and annual ring orientation of the transverse core on the mechanical performance of three-layer timber composites. A two-way analysis of variance (ANOVA) was used to assess the main effects of connection type and annual ring orientation, as well as their interaction, on the MOEapp, EIapp, MORapp, and EC.

Prior to ANOVA, the assumptions of normality of residuals and homogeneity of variances were evaluated using the Shapiro–Wilk and Levene’s tests, respectively. Pairwise comparisons between the four experimental variants were performed using Welch’s *t*-tests with Holm adjustment for multiple comparisons. Statistical significance was assessed at α = 0.05.

## 3. Results and Discussion

The physical and mechanical properties of the timber components prior to composite manufacturing are presented in Table 2. The outer lamellas exhibited a mean density of 541 kg/m^3^ and a mean modulus of elasticity of 12,953 MPa. The initial properties of the outer lamellas assigned to the four composite variants are summarized in Table 3. No statistically significant differences were observed between the groups (*p* > 0.05). These results suggest comparable initial material properties, indicating that differences in the mechanical performance of the composites were primarily associated with the interlayer connection type rather than initial timber variability. These results indicate comparable initial material properties across the variants, suggesting that the effects of connection type and annual-ring orientation on composite performance could be evaluated with only a limited confounding influence of initial timber density [22,23]. The inner layers consisted of ten individual timber elements arranged side by side. Their mean density was 544 kg/m^3^, with a standard deviation of 25 kg/m^3^. The density of the individual inner elements ranged from 378 to 661 kg/m^3^. After assembly, the mean density of the four composite beam variants ranged from 518 to 562 kg/m^3^. Variant 1 (adhesive, standing annual rings orientation) exhibited the highest mean density of 562 kg/m^3^, followed by Variant 2 (adhesive, lying annual rings orientation) at 551 kg/m^3^. The mean densities of the dowel-connected beams were 518 kg/m^3^ for Variant 3 (standing annual rings orientation) and 529 kg/m^3^ for Variant 4 (lying annual rings orientation). Overall, the density of the assembled composites remained within a relatively narrow range despite differences in connection type and annual ring orientation.

The connection type (A) had a statistically significant influence on all analysed mechanical properties (*p* < 0.001), accounting for 94.74% of the total variance in MOEapp, 87.60% in MORapp, 94.75% in EIapp, and 74.46% in EC (Table 4). The particularly high contribution to MOEapp and EIapp indicates that the method of interlayer connection was the dominant factor determining the stiffness-related behaviour of the composites. In contrast, its effect on density was not statistically significant (*p* = 0.517), with a contribution of only 0.84%. Annual ring orientation (B) had a considerably smaller influence on the analysed properties. A statistically significant effect was observed only for MOEapp (*p* = 0.0168), although its contribution to the total variance was limited to 1.22%. For the remaining properties, including density, no statistically significant effects were found (*p* > 0.05), with contributions ranging from 0.11% to 1.99%. The interaction between connection type and annual ring orientation (A × B) was statistically significant only for MOEapp (*p* = 0.0362), explaining 0.90% of its total variance. For the remaining properties, the interaction was not statistically significant (*p* > 0.05), and its contribution ranged from 0.36% to 3.87%. These findings indicate that the influence of connection type on the mechanical properties was largely independent of annual ring orientation. The residual variance was relatively low for MOEapp (3.14%), MORapp (11.86%), EIapp (3.70%), and EC (19.68%), indicating that the investigated factors explained a substantial proportion of the variability in mechanical performance. In contrast, the residual variance for density reached 95.87%, suggesting that its variability was primarily associated with factors not included in the model, particularly the natural heterogeneity of wood. Overall, the results confirm that interlayer connection type was the principal factor governing the mechanical behaviour of the composites, whereas annual ring orientation played a secondary role. The pronounced effect of connection type on MOEapp and EIapp is consistent with differences in the degree of composite action between the investigated connection systems, although the individual contributions of interlayer slip and shear deformation were not measured independently.

**Figure 5:**
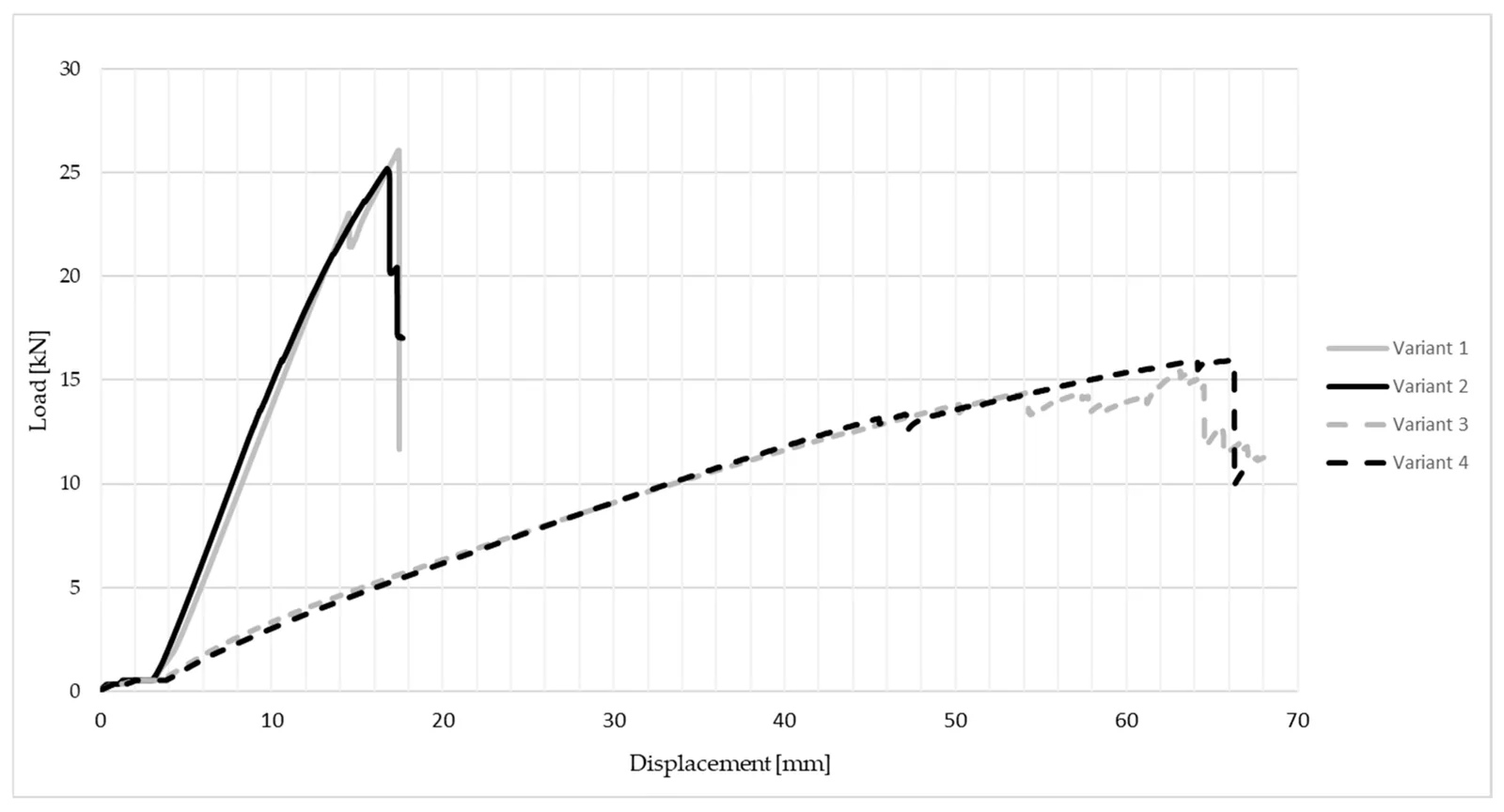
Representative load–displacement curves of the four composite configurations.

The descriptive statistics and pairwise comparisons of the investigated composite variants are presented in Table 5. The results demonstrated substantial differences in mechanical performance between adhesive-bonded and dowel-connected configurations (Figure 5). The adhesive-bonded variants exhibited significantly higher apparent global modulus of elasticity (MOEapp), apparent modulus of rupture (MORapp), and apparent bending stiffness (EIapp) compared with the dowel-connected variants.

The highest MOEapp was observed for Variant 2 (9818 MPa), which exceeded Variant 1 by approximately 17.5%. This difference was statistically significant (*p* = 0.0347), whereas the difference between the two dowel-connected variants was not significant (*p* = 0.7955). These findings suggest that the influence of annual ring orientation on global flexural response may depend on the interlayer connection system. The variability in MOEapp differed between the investigated configurations. Variant 1 exhibited a standard deviation of 1085 MPa (CV = 13.0%), compared with 87 MPa (CV = 6.4%) for Variant 4. Although the absolute variability was substantially higher for V1, the difference was less pronounced when expressed relative to the corresponding mean values. This variability may reflect the natural heterogeneity of timber and differences in deformation behaviour between the connection systems.

In contrast, the MORapp values were comparable within each connection system, indicating that the type of interlayer connection was the dominant factor influencing the nominal bending strength of the investigated composites. The apparent bending stiffness showed a similar pattern, with substantially higher values for the adhesive-bonded configurations. However, no statistically significant difference was observed between the two adhesive-bonded variants after adjustment for multiple comparisons. Energy absorption capacity exhibited a different response. The highest mean EC was recorded for Variant 4 (648 J), approximately 2.65 times that of Variant 1. This suggests that mechanically connected composites may exhibit greater deformation and energy absorption despite their lower global stiffness. Nevertheless, this interpretation should consider the potentially different contributions of interlayer slip, connector deformation, and the observed failure processes. Rostampour Haftkhani and Hematabadi [24] demonstrated the importance of layer arrangement and shear-related behaviour in determining the apparent bending properties of CLT panels. Furthermore, Ren et al. [22] highlighted the significance of connection systems and rolling shear performance in the mechanical behaviour of layered timber structures. Accordingly, the reduced apparent stiffness of the dowel-connected composites should be interpreted as the combined effect of several deformation mechanisms rather than attributed exclusively to connector compliance.

The average MOEapp of the dowel-connected composites was approximately 85% lower than that of the adhesive-bonded composites, demonstrating a pronounced reduction in global flexural stiffness associated with the discrete connection system. The observed reduction in stiffness is consistent with previous studies of adhesive-free laminated timber. Derikvand et al. [18] demonstrated that modifying the connector material can substantially improve the composite behaviour of DLT beams. Their specimens equipped with salvaged plywood tenons achieved approximately 40% greater bending stiffness than those using conventional oak dowels, which was attributed to lower end slip and a greater composite effect. This observation is particularly relevant to the present results because it indicates that the low stiffness measured for the investigated dowel-connected composites should not necessarily be regarded as an inherent limitation of all adhesive-free timber systems. Rather, it reflects the characteristics of the specific connector and connection geometry used in the study. Similarly, Xu et al. [19] investigating three-layer cross-laminated timber connected with densified-wood dowels, reported lower effective bending stiffness for dowelled elements than for conventional CLT, while also observing greater ductility of the adhesive-free system. They showed that the number and arrangement of densified-wood dowels influence the effective bending stiffness and bending strength of adhesive-free CLT. Their results demonstrated that dowelled elements can achieve useful structural performance while exhibiting greater ductility than conventional CLT.

The difference between the two connection systems can be explained by the mechanism of shear transfer between adjacent lamellae. In the adhesive-bonded specimens, the continuous adhesive layer provides distributed stress transfer along the entire interface and strongly restricts relative displacement between the lamellae. Consequently, the individual layers can participate more effectively in the bending resistance of the whole section. In the dowel-connected specimens, load transfer is concentrated at discrete connection points. Local deformation of the wood around the dowels and deformation of the dowels themselves can therefore contribute substantially to the total deflection. This reduces the degree of composite action and consequently the effective bending stiffness. However, the global deflection may also include shear deformation of the transversely oriented core layer. Since interlayer slip, connector deformation, and core shear deformation were not measured independently, their individual contributions to the apparent bending stiffness could not be quantified.

A similar difference between bonded and mechanically connected wood elements was reported by Dourado et al. [20]. In their experimental investigation of wood boards joined using structural adhesive or wood-pin connectors, the stiffness of the dowel-connected solutions was approximately half that of the bonded reference, demonstrating the importance of connection stiffness in the bending response of assembled timber members. The present results show an even more pronounced difference, with the average MOEapp of the dowel-connected elements representing only approximately 15% of that of the adhesive-bonded elements. This difference may be related to the geometry and spacing of the connectors, the stiffness of the wooden dowels used in the present study, and the limited composite action of the investigated connection. The results therefore demonstrate that the mechanical properties of the constituent pine lamellae cannot be directly transferred to the assembled element without considering the stiffness of the interlayer connection. Similar behaviour has been reported for dowel-laminated timber (DLT) and adhesive-free CLT systems, where connector compliance dominates the global deformation of the member [14,15,25].

The influence of annual-ring orientation was considerably smaller than the influence of the connection system. Changing the orientation from standing to lying increased the MOEapp of the adhesive-bonded specimens from 8353 to 9818 MPa, corresponding to an increase of approximately 18%. The relatively small contribution of annual-ring orientation to the total variance suggests that differences in radial and tangential material behaviour had a limited effect on the global flexural response under the investigated conditions. The significant interaction observed for MOEapp indicates that this effect may depend on the connection system, although the dominant influence of interlayer connection type largely determines the stiffness-related behaviour of the composites. For the dowel-connected specimens, however, the MOEapp changed only from 1395 to 1365 MPa, corresponding to a decrease of approximately 2%. Thus, although some difference between the two orientations was observed, the effect was substantially smaller than the difference resulting from the type of interlayer connection.

The two-way ANOVA confirmed a statistically significant effect of annual ring orientation (*p* = 0.0168) and its interaction with connection type (*p* = 0.0362) on MOEapp. However, their contributions to the total variance were limited to 1.22% and 0.90%, respectively, indicating that their practical importance was substantially smaller than that of connection type. Annual-ring orientation should also be distinguished from longitudinal grain direction. Its influence on the global bending response may be related to differences in radial and tangential material behaviour, although the underlying mechanisms were not independently investigated in this study. Similar conclusions have been reported for layered timber composites, in which interface shear stiffness has a much greater effect than lamella orientation [26].

A similar trend was observed for bending strength. The MORapp of the adhesive-bonded specimens was 134.4 MPa for the standing configuration and 140.8 MPa for the lying configuration. The corresponding values for the dowel-connected specimens were 73.8 and 71.6 MPa. The mean MORapp of the dowel-connected composites was therefore approximately 47% lower than that of the adhesive-bonded elements. The reduction in MORapp was considerably smaller than the reduction in MOEapp. Whereas the average stiffness decreased by approximately 85% after replacing the adhesive layer with dowels, the average bending strength decreased by approximately 47%. This indicates that the connection system affected the deformation behaviour more strongly than the ultimate bending resistance. The dowel-connected elements were therefore substantially more compliant, but they were still capable of developing a considerable proportion of the ultimate bending strength of the adhesive-bonded composites. The results are consistent with previous studies of adhesive-free timber composites. Xu et al. [19] found that dowelled CLT exhibited lower bending strength and effective bending stiffness than conventional CLT, while its greater ductility was identified as one of the characteristic advantages of the adhesive-free configuration.

A different trend was observed for energy capacity (EC). The adhesive-bonded specimens exhibited EC values of 244 and 239 J, whereas the dowel-connected specimens reached 501 and 648 J, respectively. Thus, despite their substantially lower stiffness and bending strength, the dowel-connected composites absorbed approximately 2.1–2.7 times more energy than the adhesive-bonded specimens. This result is particularly important because it demonstrates that the mechanical performance of the dowel-connected composites cannot be evaluated solely on the basis of MOEapp and MORapp. The higher EC indicates a more pronounced deformation capacity before the defined post-peak endpoint. The combination of low initial stiffness, progressive connector deformation and interlayer displacement allows the dowel-connected specimens to undergo substantially larger deformations while continuing to carry load. This behaviour is consistent with the greater ductility reported for adhesive-free, dowelled CLT by Xu et al. [19].

The higher EC of the dowel-connected specimens should therefore be interpreted together with their substantially lower apparent stiffness. The greater energy absorption may be associated with increased deformation and progressive damage rather than higher load-carrying capacity. However, the contributions of individual deformation and damage mechanisms to the total absorbed energy were not quantified. The effect of annual-ring orientation on EC was also more pronounced for the dowel-connected composites. In the adhesive-bonded specimens, EC changed from 244 to 239 J, corresponding to a difference of approximately 2%. In contrast, the lying dowel-connected configuration reached 647 J compared with 501 J for the standing configuration, representing an increase of approximately 29%. Nevertheless, the difference between Variant 3 and Variant 4 was not statistically significant, indicating that this trend should be interpreted cautiously.

The failure mechanisms differed between the adhesive-bonded and dowel-connected composites. In the adhesive-bonded specimens, failure occurred primarily within the timber, without visible damage to the adhesive bond line, suggesting that the interface maintained composite action up to failure. In contrast, the dowel-connected specimens exhibited more complex damage mechanisms, including tensile rupture of timber, local crushing around the connectors, dowel shear, and progressive interlayer displacement. The observed slip indicates partial composite action and may explain the substantially lower apparent stiffness and greater energy absorption capacity of the dowel-connected composites. In mechanically connected timber systems, shear transfer occurs through discrete connectors, whose stiffness, embedment behaviour, and fit within the surrounding wood influence the global bending response. The importance of connector characteristics for the stiffness and strength of assembled timber elements was also demonstrated by Dourado et al. [20]. However, as the individual deformation components were not measured independently and complete photographic documentation was unavailable, the relative contributions and frequency of the observed failure mechanisms could not be quantified. The observed interlayer displacement and the substantially lower MOEapp of the dowel-connected composites are consistent with partial composite action. However, the extent to which connector deformation and interlayer slip contributed to the measured deflection could not be established quantitatively.

Therefore, the effective bending response of the dowel-connected composite should be considered as the combined response of the timber lamellae and the deformable interlayer connection.

**Figure 6:**
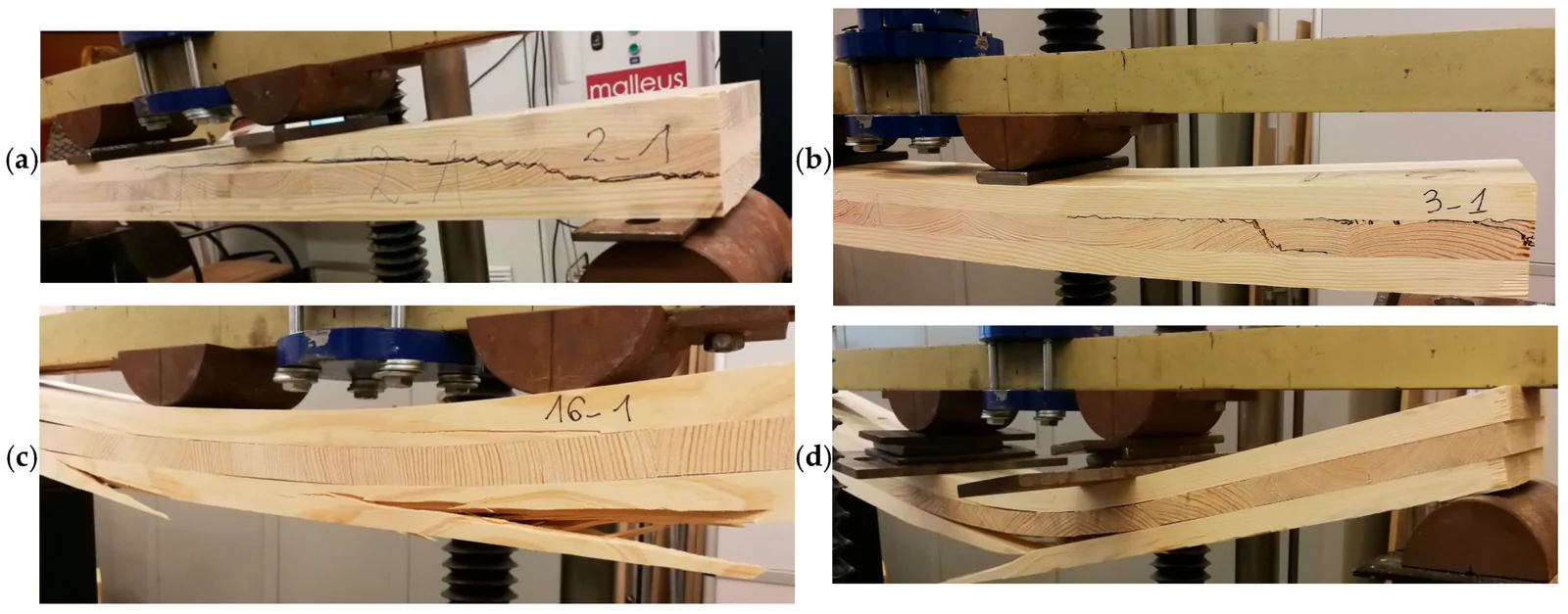
Representative failure of the three-layer timber composite under bending; (**a**) Variant 1, (**b**) Variant 2, (**c**) Variant 3, (**d**) Variant 4 of samples.

In contrast, the adhesive-bonded specimens maintained the integrity of the interlayer throughout loading. No visible failure of the adhesive bond line was observed, and failure occurred within the timber (Figure 6). This indicates that, under the conditions of the present bending tests, the adhesive interface was not the critical component of the composite. The continuous bond allowed efficient stress transfer between adjacent lamellae and maintained composite action until failure of the wood. Although no visible bond-line failure was observed, this finding alone does not demonstrate compliance with the bonding requirements applicable to structural glued timber products.

The comparison of the failure mechanisms therefore supports the mechanical results presented in Table 5. The adhesive-bonded elements combined high initial stiffness and high bending strength with relatively low deformation and energy absorption before failure. The dowel-connected elements, in contrast, exhibited much lower stiffness and strength but substantially greater energy absorption. The two connection systems consequently produced fundamentally different structural responses: the adhesive system promoted efficient load transfer and high composite action, whereas the dowel system allowed progressive deformation and slip.

The present study was limited to a single timber species, composite geometry, and wooden dowel configuration. Furthermore, connector load–slip behaviour and the individual contributions of interlayer slip, connector deformation, and transverse core shear were not measured independently. Consequently, the observed differences in apparent mechanical properties should be interpreted as the global responses of the investigated composite configurations. Future investigations should include connector-level mechanical tests, quantitative measurements of interlayer displacement, and analytical modelling of partial composite action.

## 4. Conclusions

This study investigated the effects of interlayer connection type and annual-ring orientation on the flexural performance and failure mechanisms of three-layer Scots pine composites. Based on the experimental results and statistical analysis, the following conclusions were drawn:

1. Interlayer connection type had the greatest influence on flexural performance. The dowel-connected composites exhibited approximately 85% lower apparent modulus of elasticity (MOEapp) and 47% lower apparent bending strength (MORapp) than the adhesive-bonded specimens.
2. Annual-ring orientation had a relatively limited influence on mechanical performance. Although statistically significant effects of annual-ring orientation (*p* = 0.0168) and its interaction with connection type (*p* = 0.0362) were identified for MOEapp, their contributions to the total variance were only 1.22% and 0.90%, respectively, compared with 94.74% for connection type. No statistically significant effect of annual-ring orientation was observed for MORapp or apparent bending stiffness (EIapp).
3. Dowel-connected composites exhibited greater energy absorption capacity despite their lower stiffness and strength. Their mean energy absorption was approximately 2.1–2.7 times higher than that of the adhesive-bonded specimens, indicating a greater capacity for deformation before reaching the defined post-peak failure criterion.
4. The connection systems exhibited different failure mechanisms. Adhesive-bonded specimens predominantly failed within the timber, without visible adhesive bond-line failure, whereas dowel-connected composites exhibited timber rupture, local connector-related damage, and interlayer displacement. The lower apparent stiffness of the dowel-connected specimens may reflect the combined influence of connector deformation, interlayer slip, and shear deformation of the transverse core layer. However, the individual contributions of these deformation mechanisms could not be quantified based on the measurements performed.

## Figures and Tables

**Table 1:** Variants of three-layer composite beams used in the experimental program.

Factors/Variants	Variant 1—6 Elements	Variant 2—6 Elements	Variant 3—6 Elements	Variant 4—6 Elements
Interlayer connection	PUR adhesive	PUR adhesive	Wooden dowels	Wooden dowels
Annual ring orientation of core lamellas	standing	lying	standing	lying

**Table 2:** Properties of timber components used for composite manufacturing.

Material	Configuration	Mean Density [kg/m^3^]	SD [kg/m^3^]	MOE_lam [MPa]	SD [MPa]
outer lamellas	upper and lower boards	541	38	12,953	1794
inner lamellas	elements side by side	544	25	-	-

**Table 3:** Comparison of the initial physical and mechanical properties of outer timber lamellas.

	Statistic	Variant 1	Variant 2	Variant 3	Variant 4
MOE_lam [MPa]	mean	12,441	12,596	13,708	13,065
	standard deviation	1796	2017	1378	1893
	homogeneous group	A	A	A	A
density [kg/m^3^]	mean	562	551	518	529
	standard deviation	27	30	32	9
	homogeneous group	A	A	A	A

**Table 4:** Results of the analysis of variance of the effects of interlayer connection type and annual ring on the mechanical properties of three layer timber composite.

	MOEapp		MORapp		EIapp		EC		Density	
	*p*	X [%]	*p*	X [%]	*p*	X [%]	*p*	X [%]	*p*	X [%]
Connection type (A)	<0.001	94.74	<0.001	87.60	<0.001	94.75	<0.001	74.46	0.517	0.84
Annual ring orientation (B)	0.0168	1.22	0.6067	0.18	0.5356	0.88	0.2070	1.99	0.8696	0.11
A × B	0.0362	0.90	0.4697	0.36	0.0876	0.67	0.0851	3.87	0.3777	3.18
Error		3.14		11.86		3.70		19.68		95.87

**Table 5:** Mechanical properties and density of the investigated three-layer timber composites.

	Statistic	Variant 1	Variant 2	Variant 3	Variant 4
MOEapp [MPa]	mean	8352	9817	1395	1365
	standard deviation	1085	791	203	87
	homogeneous group	B	C	A	A
MORapp [MPa]	mean	134.4	140.8	73.7	71.6
	standard deviation	10.9	16.7	7.5	13.7
	homogeneous group	B	B	A	A
EIapp [kNm^2^]	mean	23.8	27.3	4.0	3.9
	standard deviation	3.3	2.4	0.5	0.2
	homogeneous group	B	B	A	A
EC [J]	mean	244	239	501	648
	standard deviation	67	75	165	49
	homogeneous group	A	A	AB	B
Density [kg/m^3^]	mean	562	551	518	529
	standard deviation	36	29	27	10
	homogeneous group	A	A	A	A

Note: Values are presented as mean ± standard deviation (SD). Means sharing at least one letter within the same row are not significantly different according to pairwise Welch’s *t*-tests with Holm adjustment (*p* > 0.05).

## Data Availability

The original contributions presented in this study are included in the article. Further inquiries can be directed to the corresponding author.
